# Screening commercial teat disinfectants against bacteria isolated from bovine milk using disk diffusion

**DOI:** 10.14202/vetworld.2019.629-637

**Published:** 2019-05-06

**Authors:** Sarah Rose Fitzpatrick, Mary Garvey, Kieran Jordan, Jim Flynn, Bernadette O’Brien, David Gleeson

**Affiliations:** 1 Department of Livestock Systems, Teagasc, Animal and Grassland Research and Innovation Centre, Moorepark, Fermoy, County Cork, Ireland; 2 Cellular Health and Toxicology Research Group, Institute of Technology Sligo, County Sligo, Ireland; 3 Department of Food Safety, Teagasc Food Research Centre, Moorepark, Fermoy, County Cork, Ireland

**Keywords:** bacteria, disk diffusion, mastitis, teat disinfectant

## Abstract

**Background and Aim:**

Teat disinfection is an important tool in reducing the incidence of bovine mastitis. Identifying the potential mastitis-causing bacterial species in milk can be the first step in choosing the correct teat disinfectant product. The objective of this study was to screen commercial teat disinfectants for inhibition against mastitis-associated bacteria isolated from various types of milk samples.

**Materials and Methods:**

Twelve commercially available teat disinfectant products were tested, against 12 mastitis-associated bacteria strains isolated from bulk tank milk samples and bacterial strains isolated from clinical (n=2) and subclinical (n=3) quarter foremilk samples using the disk diffusion method.

**Results:**

There was a significant variation (7-30 mm) in bacterial inhibition between teat disinfection products, with products containing a lactic acid combination (with chlorhexidine or salicylic acid) resulting in the greatest levels of bacterial inhibition against all tested bacteria (p<0.05).

**Conclusion:**

In this study, combined ingredients in teat disinfection products had greater levels of bacterial inhibition than when the ingredients were used individually. The disk diffusion assay is a suitable screening method to effectively differentiate the bacterial inhibition of different teat disinfectant products.

## Introduction

Mastitis is an inflammatory process occurring in the mammary gland that can be subclinical or clinical [1], and intramammary infections (IMIs) refers to the presence of an infectious organism in the udder [2]. Mastitis can affect milk quality, animal health and welfare [3] and is one of the main milk production and economic problems facing the global dairy industry [4]. Many factors such as stage of lactation, herd size, housing and bedding type, and hygiene during milking can influence the occurrence of a new IMI [5]. The implementation of effective control programs on farms, such as teat disinfection and antimicrobial treatment of infections, is essential to reduce the effect of mastitis on the dairy industry [6,7]. Mastitis pathogens can be classified as either environmental or contagious [3], with the main mastitis-causing bacteria identified in clinical and subclinical samples in Ireland being *Staphylococcus aureus*, *Streptococcus agalactiae*, *Escherichia coli*, and *Streptococcus uberis* [1].

Teat disinfectants play an important role in a mastitis control program [8]. Previous studies have shown that both pre- and post-milking disinfection can reduce the incidence of clinical mastitis caused by contagious bacteria by 50% [9,10] and environmental bacteria by 24% [8]. Many studies have shown iodine to be effective against a wide range of mastitis-causing bacteria [8,11-13]. However, the use of iodine-based products can result in high iodine concentrations in milk, which may concern infant formula manufacturers [14]. There is now a wide range of alternative teat disinfectants available containing different active ingredients, but with limited information available on their ability to inhibit bacterial growth. Many of these teat disinfectant products contain a mixture of different ingredients. The combining of these ingredients can be problematic [7,15] as manufacturers must ensure that disinfection is maximized while skin irritation is minimized. Therefore, the active ingredient, pH, skin conditioners, and surfactant must be balanced to create an effective teat disinfectant product [15]. Bulk tank milk (BTM) analysis can determine milk quality and the presence of mastitis pathogens [16]. It can be convenient, low cost, and fast [17] and can provide information regarding on-farm hygiene conditions during milk production [18]. However, analysis of individual cow foremilk samples is more precise for monitoring udder health and pathogen diagnosis [19]. Pathogen type and numbers of bacteria in milk can differ between farms [20]. Traditional microbial and molecular methods can be used in conjunction or separately to identify mastitis-causing bacteria [21]. It is important to evaluate teat disinfectant products against mastitis-causing bacteria identified in the environment where these products will be used. The National Mastitis Council experimental challenge and natural exposure protocols are used for demonstrating field efficacy in reducing new IMI, but unfortunately, these tests can be time consuming and expensive. However, the germicidal effectiveness of teat disinfectants can be assessed using laboratory tests against a broad range of pathogens of interest [22]. At present, in the European Union, a standard method used to evaluate teat disinfectant products, before they can be sold commercially, is the BS EN 1656 [23,24]. To meet the requirements of this standard, the product tested with this standard must demonstrate at least a 10^5^ log reduction in viable counts of the test species within 5 min. The disk diffusion assay, on the other hand, has the potential to quantitatively assess the ability of a large range of teat disinfectant products to inhibit bacterial growth [25] at a laboratory scale within a short time period.

Therefore, the objective of this study was to screen commercially available teat disinfectant products by determining the ability of these products to inhibit the growth of bacteria, isolated from BTM, and subclinical and clinical quarter foremilk samples, using the disk diffusion method.

## Materials and Methods

### Ethical approval

Approval from the Institutional Animal Ethics Committee to carry out the current study was not required as no invasive procedure on the animals was performed.

### Sample collection

BTM samples were collected from 22 randomly selected dairy farms in one geographical region (Southeast of Ireland) that was willing to participate in the study. The milk was produced during October when the cows were managed outdoors and grazing on pastures. The milk samples (50 mL) were collected from the bulk tanks of these 22 dairy farms. Individual quarter foremilk samples were separately collected from clinically and subclinically infected quarters from three research farms. All of the samples were held at 4°C until analysis for bacterial identification and total bacterial counts (TBCs), which were conducted within 3-4 h of collection.

### Bacterial identification

The milk samples were serially diluted in Maximum Recovery Diluent (MRD) (Oxoid, Basingstoke, UK). Using the spread plate method, 100 μl of an appropriate dilution was transferred onto Milk Plate Count Agar (Oxoid, Basingstoke, UK) plates, which were incubated at 37°C for 24 h. 22 bacterial isolates displaying the morphology of pathogens associated with mastitis were then taken from each plate and streaked for isolation and purification. Once isolated, these bacterial cultures were Gram stained and bacterial identification was carried out using biochemical tests (catalase and oxidase tests), growth at various temperatures (25°C and 35°C), and on various types of agars [25] and using 16S rDNA sequencing of products generated by polymerase chain reaction (PCR) [21,26].

From 22 bacterial isolates, 17 bacterial isolates which displayed morphology similar to bacteria associated with mastitis were subjected to DNA extraction and PCR identification. DNA extraction was carried out using the GenElute Bacterial Genomic DNA kit (Sigma-Aldrich, Dublin, Ireland). The procedure was conducted according to the manufacturer’s instructions, with the isolated DNA samples suspended and stored in the elution solution (10 mM Tris-HCl, 0.5 mM EDTA, pH 9.0) provided in the kit. The PCR products were cleaned using AMPure XP beads. The primers used for the reaction were UniF (5′-AGAGTTTGATCCTGGCTCAGG-3′) and UniR (5′-ACGGCAACCTTGTTACGAGT-3′) and 16S rDNA gene sequencing was completed by GATC biotech (London, UK). These sequences were compared with those available in the GenBank database using the BLAST program available from the National Center for Biotechnology Information (NCBI) (http://www.ncbi.nlm.nih.gov). If species-specific sequences in the database matched the query sequence with ≥99% identity, then the query sequence was determined to come from that species [26].

TBCs were performed by serially diluting the milk samples using MRD. Then, 1 mL of the solution was pipetted onto separate Petrifilm Total Aerobic Count plates (3M, St. Paul, MN, USA), which were incubated at 32°C for 48 h and bacteria were counted using a 3M Petrifilm Plate Reader.

### Teat disinfectant information

Twelve commercially available teat disinfectant products, with different active ingredients of varying concentrations, were tested against the isolates identified above. An iodine product was chosen as a positive control, as iodine has been previously shown to effectively inhibit mastitis-related bacteria [11,25]. The disinfectants used were predominately ready-to-use products apart from two products (the control and ammonium lauryl sulfate [product number 6]) which were concentrated products requiring dilution to a usable concentration (Table-1). These products were diluted, using sterile deionized water, according to the manufacturer’s recommendation, to avoid possible issues with water hardness or contaminated water. The disinfectant products used were suitable for both pre- and post-milking teat disinfection, with the exception of two products (the control and product number 2), which were recommended only for post-milking disinfection.

**Table-1:** Test teat disinfectant product code and active ingredient, as declared by the manufacturer.

Product number	Active ingredients	Manufacturer
Control	Iodine (0.50% w/w)^1^	Agroserve, Wiltshire, United Kingdom.
1	Lactic acid (5.00% w/w) and chlorhexidine digluconate (0.30% w/w)^2^	Ark Farm Innovations, Gorey, Wexford, Ireland.
2	Chlorhexidine (0.45% w/w)^2^	Diversey Hygiene Sales Limited, Finglas, Dublin 11, Ireland.
3	Chlorhexidine digluconate (0.60% w/w)^2^	Diversey Hygiene Sales Limited, Finglas, Dublin 11, Ireland.
4	Lactic acid (0.25% w/w) and salicylic acid (Not stated)^2^	Kilco International, Dumfries and Galloway, United Kingdom.
5	Lactic acid (2.50% w/w) and chlorhexidine digluconate (0.30% w/w)^2^	Biocel Ltd., Little Island, Cork, Ireland.
6	Ammonium lauryl sulfate (3.10% w/w)^1^	Bimeda, Tallaght, Dublin, Ireland.
7	Lactic acid (1.93% w/w) and chlorhexidine digluconate (0.20% w/w)^2^	Kilco International, Dumfries and Galloway, United Kingdom.
8	Lactic acid (2.40% w/w)^2^	GEA Farm Technologies, Bönen, Germany
9	Diamine (0.60% w/w)^2^	Milk Solutions Ltd., Kilworth, Cork, Ireland.
10	Chlorine dioxide and didecyldimethylammonium chloride (0.04% w/w)^2^	Hilltop Eng. and Agri Supplies, Enniskillen, Fermanagh, N. Ireland,
11	Lactic acid (2.50% w/w) and salicylic acid (0.10% w/w)^2^	Hypred SAS, 35803 DINARD Cedex, France.

( ) Ingredients as indicated on product label,

1 Concentrate products,

2 Ready-to-use products

### Disk diffusion assay

The teat disinfectant susceptibility test was carried out using the disk diffusion assay to determine the ability of the teat disinfectant products to inhibit bacterial growth. 17 bacterial isolates from species associated with mastitis, isolated from BTM (n=12), subclinically infected quarter foremilk samples (n=3), and clinically infected quarter foremilk samples (n=2) were grown on separate Mueller-Hinton (MH) agar (Sigma-Aldrich) plates with various filter paper disks impregnated with teat disinfectant (three filter paper disks per plate).

All agars and diluents used in this study were prepared according to manufacturers’ recommendations, with 10 mL of MH agar being poured to a depth of 4 mm. The test inoculum was prepared from a pure culture grown on MH agar for 16 h. To prepare the inoculum, a loopful of the organisms was suspended in MRD and the turbidity of the suspension adjusted to a 0.5 McFarland standard (BioMerieux, UK) (approximately 10^8^ CFU/mL) [27,28]. Each bacterial suspension was used within 15 min of preparation. Using the spread plate method, 100 μl of the bacterial suspension was spread onto MH agar using a sterile “L”-shaped spreader and left to dry for 10-15 min. While drying, blank 6 mm antibiotic paper disks (Cruinn, Dublin, Ireland) were left to soak in the test disinfectant for 30 s. The disks were placed onto the agar using a sterile forceps, ensuring the entire disk was touching the agar. The plates were then incubated at 37°C for 24 h. After incubation, the zones of inhibition were measured using an electronic caliper (Mitutoyo Digimatic, RS digital caliper 600/880).

After completion of the disk diffusion test on the 12 teat disinfectant products, specific products which resulted in large zones of inhibition and small zones of inhibition were subjected to testing under the BS EN 1656 standard to determine if those products met the required 10^5^ log reduction.

### Statistical analysis

Twelve bacterial isolates from BTM samples found to be associated with mastitis and five mastitis-causing bacterial isolates from quarter foremilk samples (subclinically infected quarter foremilk samples [n=3] and clinically infected quarter foremilk samples [n=2]) were used in the study. The experiments were independently repeated on three occasions, with three plate replicates for each experimental data point giving a mean result for each experimental batch.

Statistical analysis was performed using SAS for Windows, version 9.4(SAS Institute Inc., Cary, NC, USA). The results were analyzed using PROC GLIMMIX. Pair-wise comparisons were adjusted for multiplicity effect using simulation procedures to adjust P-values. Residual checks were made to ensure that assumptions of the analysis were met. The LSMEANS statement in PROC GLIMMIX was used to differentiate statistical differences. The zones of inhibition for the bacteria isolated from BTM and the bacteria isolated from quarter foremilk samples were analyzed using two models (one for each milk sample source). Both models included the zones of inhibition as a dependent variable and product and bacteria as independent variables.

## Results

The 12 mastitis-associated isolates, identified from BTM samples using biochemical tests and 16S rDNA sequencing, were placed into one of two groups: (1) Environmental bacteria which included; *Enterococcus faecalis*, *Hafnia alvei*, *Serratia marcescens*, *Serratia liquefaciens*, *Aerococcus viridans*, and *Lactococcus lactis* and (2) staphylococcal bacteria which included; *Staphylococcus chromogenes*, *Staphylococcus devriesei*, *Staphylococcus epidermis*, *Staphylococcus haemolyticus*, *Staphylococcus hominis*, and *Staphylococcus xylosus* (Table-2).

**Table-2:** Mastitis-associated bacterial isolates identified from BTM samples with isolate numbers and TBC for each BTM sample.

Bacterial species	BTM sample code (number of isolates)	TBC (CFU/mL)
Environmental		
*Aerococcus viridans*	52 (1)	2,000
*Enterococcus faecalis*	26 (1)	465,000
*Hafnia alvei*	33 (1)	29,000
*Lactococcus lactis*	45 (1)	7,050
*Serratia liquefaciens*	10 (1)	9,550
*Serratia marcescens*	4 (1)	78,500
Staphylococcal species		
*Staphylococcus chromogenes*	10 (1)	9,550
*Staphylococcus devriesei*	5 (1)	4,400
*Staphylococcus epidermis*	55 (1)	67,000
*Staphylococcus haemolyticus*	14 (1)	4,350
*Staphylococcus hominis*	55 (1)	67,000
*Staphylococcus xylosus*	22 (1)	500

Total number of isolates associated with mastitis=12. BTM=Bulk tank milk, TBC=Total bacterial count

From the different clinical and subclinical samples, five mastitis-associated isolates were identified. Three *S. aureus* strains (*S*. *aureus* 324, *S*. *aureus* 308, and *S*. *aureus* 311) were isolated from subclinical quarter foremilk samples. In addition, one *S*. *uberis* and one *E*. *coli* were identified in clinical quarter foremilk samples.

### Inhibiting the growth of bacteria identified from BTM samples

The disk diffusion method established the ability of the teat disinfectant products to inhibit the growth of the bacterial species identified from BTM and quarter foremilk samples. The TBC for BTM ranged from 500 to 465,000 CFU/mL, with farms with high TBC values (>98,517 CFU/mL) having more environmental bacteria than the farms with lower TBC values. The farms in which the described staphylococcal species were recovered had an overall average TBC of 17,070 CFU/mL (Table-2).

#### Environmental bacterial group

Within the group of environmental bacteria, *H*. *alvei*, *S*. *liquefaciens*, and *S*. *marcescens* were the most resistant bacteria on average across all teat disinfectants, while *L*. *lactis* was the most susceptible bacteria to all tested teat disinfectants (p<0.05). Overall, the product containing a combination of lactic acid and salicylic acid (product number 11) had the greatest bacterial inhibitions against all environmental bacteria. This product resulted in an overall average (±SE) zone of inhibition of 21.3 (±0.65) mm. In comparison, the control resulted in an overall average zone of inhibition of 15.5 (±0.46) mm, which was less than lactic acid and salicylic acid (product 11) (p<0.05). The zones of inhibition of teat disinfectant products compared to the iodine control against each environmental bacterial strain are shown in Table-3. The product containing ammonium lauryl sulfate (product number 6) had significantly smaller results of bacterial inhibition against environmental bacteria compared to all teat disinfectants, resulting in an average zone of inhibition of 11.8 (±0.43) mm across all environmental bacteria (Table-3).

**Table-3:** Average (±SE) zone of inhibition (mm) for each environmental bacterial species isolated from BTM samples against each teat disinfectant.

Bacteria	Teat disinfectant (average zones of inhibition [mm])											

	Cont.	1	2	3	4	5	6	7	8	9	10	11
EFA	19^a^ (0.4)	24^b^ (0.3)	16^a^ (0.4)	18^a^ (0.4)	21^a^ (0.4)	22^a^ (0.4)	14^b^ (0.3)	20^a^ (0.8)	19^a^ (0.4)	12^b^ (0.3)	15^b^ (0.6)	22^b^ (0.6)
HAL	11^a^ (0.3)	18^b^ (0.2)	16^b^ (0.5)	14^a^ (0.3)	13^a^ (1.2)	20^b^ (0.6)	7^b^ (0.3)	16^b^ (0.8)	12^a^ (1.1)	14^a^ (0.5)	16^b^ (0.9)	21^b^ (1.2)
SMA	14^a^ (0.4)	19^b^ (0.3)	15^a^ (0.5)	15^a^ (0.4)	14^a^ (0.7)	19^b^ (0.4)	8^b^ (0.1)	15^b^ (0.7)	12^a^ (1.1)	16^b^ (0.7)	8^b^ (1.4)	20^b^ (0.4)
SLI	11^a^ (0.3)	19^b^ (0.3)	16^b^ (0.3)	16^b^ (0.3)	13^a^ (0.5)	20^b^ (0.4)	7^b^ (0.2)	14^b^ (0.5)	11^a^ (0.5)	15^b^ (0.6)	16^b^ (0.4)	19^b^ (0.6)
AVI	17^a^ (0.3)	21^b^ (0.4)	18^a^ (0.4)	19^a^ (0.5)	20^b^ (0.5)	20^b^ (0.5)	14^b^ (0.4)	20^b^ (0.5)	19^a^ (0.3)	16^a^ (0.5)	15^a^ (0.3)	21^b^ (0.4)
LLA	21^a^ (1.1)	22^a^ (1.1)	21^a^ (0.7)	20^a^ (0.5)	23^a^ (1.2)	22^a^ (0.2)	21^a^ (1.3)	25^b^ (0.5)	22^a^ (0.4)	19^a^ (0.6)	18^a^ (0.2)	25^a^ (0.7)

a, b Denotes significant difference (p<0.05) between the control and the teat disinfectants against each isolated bacterial species within rows. Teat disinfectant: Cont.=Control 0.5% w/w iodine, 1=Lactic acid and chlorhexidine, 2=Chlorhexidine, 3=Chlorhexidine, 4=Lactic acid and salicylic acid, 5=Lactic acid and chlorhexidine, 6=Ammonium lauryl sulfate, 7=Lactic acid and chlorhexidine, 8=Lactic acid, 9=Diamine, 10=Chlorine dioxide, and 11=Lactic acid and salicylic acid. Bacteria: SMA=*Serratia marcescens*, SLI=*Serratia liquefaciens*, EFA=*Enterococcus faecalis*, HAL=*Hafnia alvei*, LLA=*Lactococcus lactis*, AVI=*Aerococcus viridans*. BTM=Bulk tank milk

#### Staphylococcal species group

Within the group of staphylococcal bacteria, *S*. *haemolyticus*, *S*. *Xylosus*, and *S*. *devriesei* were the most resistant bacteria, while *S*. *hominis*, *S*. *Epidermis*, and *S*. *chromogenes* were the most susceptible bacteria. Overall, within the group of staphylococcal bacteria, the product which contained a combination of lactic acid and salicylic acid (product number 11) resulted in the largest zones of inhibition (average zones of inhibition of 25.3 [±0.85] mm) in comparison to the control which had an overall average zone of inhibition of 17.7 (±0.63) mm for all staphylococcal bacteria (p<0.05). The zones of inhibition of test teat disinfectant products compared to the iodine control for each staphylococcal bacterial strain are displayed in Table-4. However, the product containing ammonium lauryl sulfate resulted in smaller zones of inhibition, resulting in an overall average zone of inhibition of 15.8 (±0.52) mm (Table-4). Overall, environmental bacteria were more resistant than staphylococcal bacteria to all teat disinfectant products (p<0.05).

**Table-4:** Average (±SE) zone of inhibition (mm) for each staphylococcal bacterial species isolated from BTM samples against each teat disinfectant.

Bacteria	Teat disinfectant (average zones of inhibition [mm])											

	Cont.	1	2	3	4	5	6	7	8	9	10	11
SDE	17^a^ (0.9)	24^b^ (0.9)	18^a^ (0.9)	19^a^ (0.7)	21^b^ (1.0)	23^b^ (1.1)	15^a^ (0.6)	19^a^ (0.7)	19^a^ (0.7)	22^b^ (0.9)	15^a^ (0.6)	25^b^ (1.4)
SHO	17^a^ (0.7)	24^b^ (0.3)	27^b^ (0.8)	28^b^ (0.2)	21^b^ (1.1)	29^b^ (0.5)	18^a^ (0.3)	28^b^ (0.6)	22^b^ (0.8)	24^b^ (0.9)	29^b^ (1.1)	30^b^ (0.9)
SEP	18^a^ (0.3)	22^a^ (1.2)	24^b^ (1.1)	24^b^ (0.9)	24^a^ (1.0)	24^b^ (0.5)	17^b^ (0.5)	26^b^ (0.7)	22^a^ (0.8)	21^a^ (1.1)	28^b^ (1.4)	29^b^ (0.9)
SCH	19^a^ (0.3)	23^b^ (0.5)	21^a^ (0.5)	20^a^ (0.2)	22^a^ (0.5)	24^b^ (0.4)	18^a^ (0.4)	23^b^ (0.4)	21^a^ (0.5)	18^a^ (0.6)	21^a^ (0.6)	22^a^ (0.7)
SHA	18^a^ (0.7)	19^a^ (0.5)	18^a^ (0.4)	19^a^ (0.3)	15^b^ (0.5)	20^a^ (0.5)	13^b^ (0.6)	18^a^ (0.7)	18^a^ (0.5)	19^a^ (0.3)	20^a^ (0.3)	22^a^ (0.7)
SXY	17^a^ (0.9)	23^b^ (1.3)	20^a^ (0.4)	18^a^ (0.3)	18^a^ (1.6)	23^b^ (1.0)	14^b^ (0.7)	20^a^ (0.3)	17^a^ (1.2)	19^a^ (0.3)	18^a^ (0.4)	24^b^ (0.5)

a, b Denotes significant difference (p<0.05) between the control and the teat disinfectants against each isolated bacterial species within rows. Teat disinfectant: Cont.=Control 0.5% w/w iodine, 1=Lactic acid and chlorhexidine, 2=Chlorhexidine, 3=Chlorhexidine, 4=Lactic acid and salicylic acid, 5=Lactic acid and chlorhexidine, 6=Ammonium lauryl sulfate, 7=Lactic acid and chlorhexidine, 8=Lactic acid, 9=Diamine, 10=Chlorine dioxide, and 11=Lactic acid and salicylic acid. Bacteria: SDE=*Staphylococcus devriesei*, SCH=*Staphylococcus chromogenes*, SHA=*Staphylococcus haemolyticus*, SXY=*Staphylococcus xylosus,* SEP=*Staphylococcus epidermis,* SHO=*Staphylococcus hominis*. BTM=Bulk tank milk

### Inhibiting the growth of bacteria identified from clinical and subclinical quarter foremilk samples

The comparison of test teat disinfectant products to the iodine control for the quarter foremilk isolates is shown in Table-5. *E*. *coli* was the most resistant bacteria with *S. uberis* being the most susceptible to the teat disinfectants tested (p>0.05).

**Table-5:** Average (±SE) zone of inhibition (mm) for each isolated bacterial species from quarter foremilk samples against each teat disinfectant.

Bacteria	Teat disinfectant (average zones of inhibition [mm])											

	Cont.	1	2	3	4	5	6	7	8	9	10	11
*Staphylococcus aureus* 324	18^a^ (0.8)	20^a^ (0.8)	21^a^ (0.5)	19^a^ (0.7)	16^a^ (0.5)	20^a^ (0.4)	15^a^ (0.5)	21^b^ (0.5)	17^a^ (0.4)	14^b^ (0.5)	25^b^ (1.3)	19^a^ (0.6)
*Staphylococcus aureus* 308	18^a^ (0.7)	20^b^ (0.5)	21^b^ (0.5)	19^a^ (0.6)	16^a^ (0.5)	19^a^ (0.5)	16^a^ (0.5)	20^a^ (0.6)	19^a^ (0.7)	15^b^ (0.5)	24^b^ (0.9)	19^a^ (0.4)
*Staphylococcus aureus* 311	18^a^ (0.7)	20^a^ (0.5)	21^a^ (0.7)	20^a^ (1.1)	15^a^ (0.5)	20^a^ (0.6)	15^b^ (0.5)	20^a^ (0.4)	18^a^ (0.6)	15^b^ (0.5)	24^b^ (1.1)	20^a^ (0.7)
*Escherichia coli*	14^a^ (1.2)	19^a^ (0.6)	18^a^ (0.4)	17^a^ (0.5)	14^a^ (0.8)	20^a^ (0.7)	12^b^ (1.6)	17^a^ (0.4)	13^a^ (1.3)	16^a^ (0.8)	13^a^ (1.3)	19^a^ (0.5)
*Streptococcus uberis*	20^a^ (0.6)	24^b^ (0.7)	22^a^ (0.6)	21^a^ (0.7)	21^a^ (0.4)	22^a^ (0.6)	16^b^ (0.3)	23^b^ (0.7)	21^a^ (0.5)	17^b^ (0.4)	18^a^ (0.9)	25^b^ (0.9)

a, b Denotes significant difference (p<0.05) between the control and the teat disinfectants against each isolated bacterial species within rows. Teat disinfectant: Cont.=Control 0.5% w/w iodine, 1=Lactic acid and chlorhexidine, 2=Chlorhexidine, 3=Chlorhexidine, 4=Lactic acid and salicylic acid, 5=Lactic acid and chlorhexidine, 6=Ammonium lauryl sulfate, 7=Lactic acid and chlorhexidine, 8=Lactic acid, 9=Diamine, 10=Chlorine dioxide, and 11=Lactic acid and salicylic acid.

**Figure-1:**
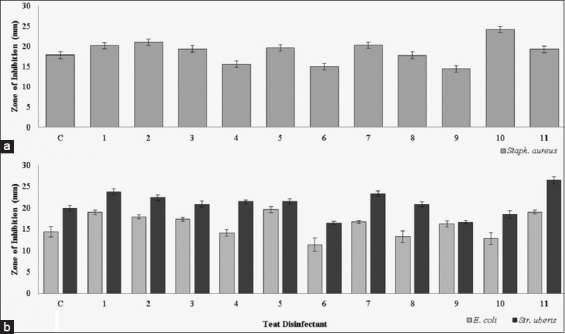
The average zone of inhibition (mm) for teat disinfectant products against bacteria isolated from (a) subclinical quarter foremilk samples (average of the three *Staphylococcus aureus* isolates) and (b) clinical quarter foremilk samples (*Escherichia coli* and *Staphylococcus* *uberis*). Error bars indicate SE. Teat disinfectant: C=Control (0.5% iodine), 1=Lactic acid and chlorhexidine, 2=Chlorhexidine, 3=Chlorhexidine, 4=Lactic acid and salicylic acid, 5=Lactic acid and chlorhexidine, 6=Ammonium lauryl sulfate, 7=Lactic acid and chlorhexidine, 8=Lactic acid, 9=Diamine, 10=Chlorine dioxide, and 11=Lactic acid and salicylic acid.

The zones of inhibition of teat disinfectant products were not significantly different between the three different *S*. *aureus* isolates identified from subclinical quarter foremilk samples (p>0.05). Products containing chlorine dioxide (product number 10) and chlorhexidine (product number 2) resulted in the largest zones of inhibition for all three isolates of *S*. *aureus*. These products resulted in average zones of inhibition of 24.3 (±1.10) and 21.0 (±0.56) mm, respectively. In comparison, diamine (product number 9) had the smallest result of bacterial inhibition for all three isolated *S*. *aureus*, resulting in an average zone of inhibition of 15.0 (±0.50) mm. The average zones of inhibition across all three *S*. *aureus* strains are presented in Figure-1a.

The products with the greatest amount of bacterial inhibition against *E*. *coli* contained a combination of lactic acid and chlorhexidine (product number 5) and lactic acid and salicylic acid (product number 11). These products resulted in the greatest average zones of inhibition of 20.0 (±0.70) and 19.0 (±0.51) mm, respectively. In comparison, a product containing ammonium lauryl sulfate (product number 6) resulted in an average zone of inhibition of 12.0 (±1.60) mm against *E*. *coli* (Table-5).

Lactic acid and salicylic acid (product number 11) were the most effective product against *S*. *uberis*. This product resulted in an average zone of inhibition of 25.0 (±0.90) mm, in comparison to the control, which showed an average zone of inhibition of 20.0 (±0.6) mm (p<0.05). Similarly, to *E*. *coli* and *S*. *aureus* isolates, a product containing ammonium lauryl sulfate (product number 6) was the least effective against *S*. *uberis*, resulting in an average zone of inhibition of 16.0 (±0.30) mm, compared to all of the teat disinfectants tested (p<0.05) (Table-5). The zones of inhibition for *E*. *coli* and *S*. *uberis* are presented in Figure-1b.

### Evaluation of products using the BS EN 1656 standard

At the conclusion of the disk diffusion study, two teat disinfectant products were selected for evaluation using the BS EN 1656 standard. A disinfectant that generally produced large zones of inhibition (product 1) and one that was typically resulted in smaller zones (product 9) was selected for evaluation. Products 1 and 9 had log reductions greater than that required for this standard (10^5^).

## Discussion

Of the bacteria isolated from BTM samples, environmental bacteria were more resistant to all teat disinfectant products than staphylococcal isolates, which is similar to other studies [8,29]. A previous study showed that teat disinfectant products (hydrogen peroxide), chlorine dioxide, 1% w/w iodophor, and 0.5% w/w iodophor) resulted in higher log reductions against staphylococcal isolates than *E*. *coli* [29].

Ten of the teat disinfectant products tested resulted in higher levels of bacterial inhibition against the bacteria isolated from BTM compared to the control. The majority of teat disinfectant products were significantly different from each other (p<0.05), with products containing a mix of lactic acid (2.5-5% w/w) and chlorhexidine (0.3% w/w) (product numbers 1 and 5) and a mix of lactic acid (2.5% w/w) and salicylic acid (0.1% w/w) (product number 11) resulting in the greatest levels of bacterial inhibition against both environmental and staphylococcal species bacteria. The previous studies have shown these ingredients to be effective in the control of IMIs. A chlorhexidine product (0.35% w/w) was previously shown to reduce IMIs caused by major mastitis bacteria by 50% compared to non-disinfected teats [30]. Lactic acid, when used as an activator to create a chlorous acid post-milking teat disinfectant, was shown to reduce new IMIs caused by *S*. *aureus* and *S*. *agalactiae* by 69% and 35%, respectively [31]. Similarly, a sodium chlorite-lactic acid-based teat disinfectant had a higher percent log reduction compared to an iodine-based teat disinfectant [32].

The bacteria isolated from individual quarter foremilk samples were similar to those identified in a previous study that created a list of bacterial pathogens responsible for clinical and subclinical mastitis in dairy herds in Ireland over a full lactation [1]. The most common mastitis-causing pathogens identified from quarter foremilk samples were *E*. *coli*, *S*. *aureus*, *S*. *uberis*, *Streptococcus* species, and coagulase-negative *Staphylococci*, with *S*. *aureus* accounting for 60% of all mastitis cases [1].

A difference in teat disinfectant ability to inhibit the growth of bacteria was observed against all bacterial species isolated from clinical and subclinical quarter foremilk samples, with *E*. *coli* being the most resistant bacteria to the teat disinfectant products tested. Similarly, a wide range of teat disinfectants (iodine, chlorhexidine, sodium hypochlorite, quaternary ammonium, bronopol, and iodophor) resulted in significantly lower log reductions for *E*. *coli* and high log reductions for *S*. *aureus* [33]. In this study, lactic acid combined with salicylic acid (product number 11) resulted in the largest zones of inhibition against both *E*. *coli* and *S*. *uberis*. Similarly, a sodium chlorite-lactic acid teat disinfectant was highly effective against *E*. *coli* [32]. However, chlorine dioxide (product number 10) resulted in large zones of inhibition against all three *S*. *aureus* isolates, compared to the control. Chlorine dioxide has been shown to be one of the most effective products against *S*. *aureus* [29] and most effective in pre-milking cleaning regimes [29,34,35]. A chlorine dioxide teat disinfectant was also found to be comparable to an iodine-based teat disinfectant [36].

For both BTM and quarter foremilk sample isolates, products containing 5% w/w lactic acid and 0.3% w/w chlorhexidine (product number 1), 2.5% w/w lactic acid and 0.3% w/w chlorhexidine (product number 5), and 2.5% w/w lactic acid and 0.1% w/w salicylic acid (product number 11) may have achieved large zones of inhibition due to the levels of active ingredient. This may be due to the high concentration and combination of the active ingredients as a product containing a lower lactic acid content (0.25% w/w) (product number 4), resulted in smaller zones of inhibition compared to these products. This may suggest that the levels of active ingredients as applied in the products and when active ingredients are combined rather than when used individually may result in better bacterial inhibition. A previous study found that 0.1% w/w chlorhexidine teat disinfectant was not effective against *S*. *aureus* and *S*. *agalactiae*, due to a low germicide concentration and recommended the use of 0.5% w/w chlorhexidine [37]. However, 0.35% w/w chlorhexidine teat disinfectant resulted in over a 50% reduction of *S*. *aureus* [30]. Furthermore, products containing one active ingredient, such as chlorhexidine (product numbers 2 and 3) or lactic acid (product number 8), did not attain large zones of inhibition as products with combined active ingredients.

The disk diffusion assay used in this study has previously been used to evaluate a range of antibiotics [38,39] and a teat disinfectant product of varying concentration [25]. Procedures recommended by CLSI [28], for the application of this method, were used and adapted in this study to determine the bacterial inhibition of a wide range of teat disinfectants. The disk diffusion method can differentiate the bacterial inhibition of teat disinfectant products, whereas the BS EN 1656 aims to achieve at least a 10^5^ log reduction. The teat disinfectant products used in this study are commercially available and have all previously undergone testing using the BS EN 1656 standard and would have achieved the required log reduction. In this study, two products (one with small and one with large zones of inhibition) were also evaluated using BS EN 1656, and both products achieved the required log reduction.

The disk diffusion method allows for laboratory screening of a large number of teat disinfectant products within a short time period against a bacterial species or strain of interest. Unfortunately, this method is limited in how it tests the product as it does not take into account contact time of the teat disinfectant, the reduction of the bacterial load on the teat skin surface, or the products true efficacy in reducing the number of new IMIs. In comparison, naturally occurring and experimental challenge methods can test the efficacy of a teat disinfectant product as the reduction of new IMIs and the reduction of teat skin bacteria can be recorded. However, there is considerable expense and time associated with such studies.

The results showed that there is a range of alternative teat disinfectant products available which reduce bacterial growth comparable to iodine-based products. Field trials would be required to fully determine the products efficacy and ability to reduce IMIs.

## Conclusion

This study gives an indication of the ability of teat disinfectant products to inhibit the growth of mastitis-causing bacteria identified in Irish milk samples. The three products which had the greatest levels of bacterial inhibition evaluated against the most prevalent mastitis-causing bacteria (*S. aureus* and *S*. *uberis*) in Irish herds contained a combination of lactic acid (2.5 or 5% w/w) and chlorhexidine (0.3% w/w) or salicylic acid (0.1% w/w). This may suggest that some active ingredients may work more successfully when combined with other active ingredients, rather than when used individually. The levels of lactic acid in combination products may also be important as levels of 2.5% w/w, or greater, showed greater bacterial inhibition compared to products with lower levels of lactic acid. The disk diffusion assay is a suitable screening method to effectively differentiate the bacterial inhibition of different teat disinfectant products.

## Authors’ Contributions

SRF and DG designed and supervised the study. SRF analyzed the samples. SRF drafted the manuscript. DG, MG, KJ, JF, and BOB discussed and edited the manuscript. All authors read and approved the final manuscript.
